# 3D-Visualization of Neurovascular Compression at the Ventrolateral Medulla in Patients with Arterial Hypertension

**DOI:** 10.1007/s00062-020-00916-z

**Published:** 2020-05-27

**Authors:** Panagiota Manava, Ramin Naraghi, Roland Schmieder, Rudolf Fahlbusch, Arnd Doerfler, Michael M. Lell, Michael Buchfelder, Peter Hastreiter

**Affiliations:** 1 Department of Neurosurgery, Friedrich-Alexander University, Erlangen-Nuremberg (FAU), Erlangen, Germany; 2 Department of Nephrology and Hypertension, Friedrich-Alexander University, Erlangen-Nuremberg (FAU), Erlangen, Germany; 3 Division of Neuroradiology, Friedrich-Alexander University, Erlangen-Nuremberg (FAU), Erlangen, Germany; 4 International Neuroscience Institute, Hannover, Germany; 5 Department of Radiology and Nuclear Medicine, Paracelsus Medical University, Nuremberg, Germany; 6 Department of Neurosurgery, German Armed Forces Central Hospital Koblenz, Koblenz, Germany

**Keywords:** Cranial nerves, Image fusion, MRI, Image processing, Posterior fossa, Vascular compression disorders

## Abstract

**Purpose:**

Controversy exists on the association of arterial hypertension (HTN) and neurovascular compression (NVC) at the ventrolateral medulla (VLM). No standardized and reproducible technique has been introduced yet for detection of NVC in HTN. This study aimed to generate, analyze and compare different results of exact reproducible anatomical 3D-representations of the VLM in patients with HTN, based on magnetic resonance imaging (MRI).

**Methods:**

A 3T scanner provided MRI (T2-constructive interference in steady state (CISS) high resolution imaging and three-dimensional Time-of-flight (3D-TOF) angiography) from the posterior fossa of 44 patients with clinical treatment-resistant HTN. Image processing consists of segmentation of the CISS data, registration and fusion of the CISS and TOF data and visualization. For each patient two 3D-visualizations (before and after fusion) were obtained. The reproduction quality of the vessels, flow-related signal variability and pulsation artifacts were analyzed and compared, using a ranking score.

**Results:**

Integrating vascular information from TOF into CISS data reduced artifacts in 3D-visualizations of exclusively processed CISS data. The quality of 3D-visualization of the vessels near the brain stem was significantly improved (*p* = 0.004). The results were reproducible and reliable. The quality of the 3D-presentations of neurovascular relationships at the VLM improved significantly (*p* < 0.001).

**Conclusion:**

The 3D-visualization of fused image data provides an excellent overview of the relationship between cranial nerves and vessels at the VLM and simplifies the detection of NVC in HTN. It provides a powerful tool for future clinical and scientific research. Although microvascular decompression (MVD) in treatment resistant HTN is not a standard procedure, it can be discussed in selected patients with intractable severe HTN.

## Introduction

Jannetta et al. [1] suggested an association between essential hypertension (HTN) and neurovascular compression (NVC) at the ventrolateral medulla (VLM) [1]. In 1985 they published the results after microvascular decompression (MVD) of the VLM in a series of 42 patients suffering from HTN. He reported a significant reduction of blood pressure up to normalization in 75% almost corresponding to success rates of other hyperactive cranial nerve dysfunctions [1]. This possible association between HTN and NVC was then supported by autopsy [2] and magnetic resonance imaging (MRI) findings [3–10]. A total of three distinct types of NVC at the VLM in HTN were identified [2]. The development of high-resolution MRI techniques enables the details of small structures within the cisterns of the posterior fossa to be examined. Established sequences for preoperative assessment of NVC are high spatial resolution strong 3D-gradient echo T2-weighted imaging, i.e. constructive interference in steady state (CISS) and MR-angiography, i.e. time-of-flight (TOF) [11–15]. In addition, the higher signal of 3 T can improve the spatial resolution for the assessment of NVC [16].

In order to create comprehensible anatomical representations of NVC in patients with typical hyperactive cranial nerve dysfunction syndromes, such as trigeminal neuralgia or hemifacial spasm a strategy for processing and visualization of medical images consisting of three consecutive steps (segmentation, registration and fusion, visualization) was introduced [15, 17]. The results of the visualizations correlate well with surgical findings [7].

Major problems limiting the quality of 3D-visualizations are flow-related signal variability of vessels, pulsation artifacts of the cerebrospinal fluid (CSF) in the source image data and the fusion of the contours from vessels very close to the brainstem [7].

The aim of this study was to introduce by comparison of different approaches a standard, robust and reproducible optimized method of 3D-visualization for the relationship between vessels and cranial nerves (CN) at the VLM in patients with HTN.

In previous studies the effectiveness of 3D-visualization of NVC in typical compression syndromes like trigeminal neuralgia and hemifacial spasm could be demonstrated. The detection of NVC at the VLM in HTN can be the first step towards a possible MVD for this entity.

## Material and Methods

### Clinical Data

The ethics committee of the University approved this study. Informed consent was obtained from all patients explaining the prospective research character of the study. 44 patients (8 women, 36 men, age range 32–70 years, mean 57 years) with treatment resistant HTN from the Department of Nephrology and Hypertension were recruited for this study.

Treatment resistant HTN was defined as office BP ≥140/90 mm Hg (mean of 2 measurements), while treated with at least 3 antihypertensive drugs, 1 of which was a diuretic [18]. True resistant HTN was confirmed by 24‑h ambulatory BP measurements (average 24‑h BP ≥130/80 mm Hg). All patients were consecutively enrolled and referred to the university outpatient service to rule out secondary causes of HTN.

An NVC is defined as one of the different types of loop (I–III) according to Naraghi et al. [2]. A positive finding of NVC results from a close contact at the VLM level between the vessel and the root entry zone (REZ) of cranial nerves (CN) IX and X, whereby one of the different types of vascular loops has to be present at the same time. In this study no MVD was performed and the treatment of HTN was not modified based on the results.

### Magnetic Resonance Imaging

All scans were obtained with a 3.0 T MR system (Magnetom Trio, Siemens Healthcare, Erlangen, Germany). Two sequences CISS (acquisition time 8:26 min:s, voxel size 0.4 mm^3^, sections per slab: 144, matrix 512 × 512, TR 7.48 ms, TE 3.23 ms, flip angle 45°) and TOF (acquisition time 15:51 min:s, voxel size 0.4 mm^3^, sections per slab 144, matrix 512 × 512, TR 21 ms, TE 3.77 ms, flip angle 18°) were used. CISS provides images with a high degree of contrast between the hyperintense cerebrospinal fluid (CSF) and the hypointense cranial nerves, brainstem and vessels. TOF is the most commonly used MR angiography sequence in neurovascular imaging with the highest resolution. Using signals of floating fluid it displays the vessels as hyperintense structures without using a contrast agent [19]. Both sequences were adjusted to isotropic voxel size (0.4 mm) ensuring equally high resolution in axial, coronal and sagittal slice images.

The vessels of interest at the VLM are vertebral artery (VA), basilar artery (BA), left and right posterior inferior cerebellar artery (PICA) and anterior inferior cerebellar artery (AICA). The following nerves were visualized: trigeminal nerve (CN V), facial nerve (CN VII), vestibulocochlear nerve (CN VIII), glossopharyngeal nerve (CN IX), vagus nerve (CN X) and accessory nerve (CN XI).

### Image Processing

For the visual representation of the MR data, a previously introduced approach based on direct volume rendering was applied [7, 15, 20, 21].

It was fully integrated into the extensible software framework medical analysis and visualization (MedAlyVis, developed in the University of Erlangen), which was developed at the Chair for Computer Graphics and at the Neurocenter of the Department of Neurosurgery, both at the University of Erlangen-Nuremberg. The framework was developed for clinical experts and is intended to support communication between physicians and software engineers. Based on the digital imaging and communications in medicine (DICOM) standard image data can be imported from and exported to Picture Archiving and Communication System (PACS). The applied method enables a clear and easy differentiation of the neurovascular structures in CISS data. Since the cranial nerves, the vessels, the brainstem and the surrounding tissues are in the same range of low intensity values in CISS data, it is impossible to separate them by simple assignment of color and opacity values with a conventional approach of direct volume rendering. In contrast, an explicit and detailed segmentation of the tiny nerve and vessel structures would be very time consuming and prone to errors.

In order to obtain a meaningful 3D-visualization of the nerve and vessel structures, the applied method combines coarse explicit and detailed implicit segmentation. Explicit segmentation explicitly assigns every voxel of the image data to a specific anatomical structure and implicit segmentation in the context of direct volume rendering assigns individual look-up tables for color and opacity to each anatomical structure. In the beginning, the method uses a sequence of preprocessing operations for noise reduction (anisotropic diffusion) and morphological filtering with a 3D-grey value closing operation. This leads to the removal of hypointense signals within the hyperintense CSF. As a result, the CSF becomes a more compact body that can be extracted from the surrounding structures. Then, explicit segmentation based on volume growing was used to separate the CSF space of the basal cistern ventral and lateral to the brainstem including all nerves and vessels. For volume growing, upper and lower thresholds, which were selected close to the signal intensity of CSF, were used to limit the range of intensity values. Additionally, a bounding box helped to restrict the volume of interest. The segmented closed CSF volume is used as a mask for labeling the original MR-CISS data during volume rendering. In the next step, the brainstem was delimited with volume growing by using the previously segmented CSF area as well as a simple lateral and dorsal restriction with a bounding box as a boundary.

In the final step, the cranial nerves, which are represented in the same range of intensity values as vessels in CISS data, were segmented. This was achieved manually since it requires in-depth anatomical knowledge. As a result, the steps of explicit segmentation divided the CISS data into four sub-volumes according to the respective anatomical structures: (1) CSF area including all vascular structures, (2) brainstem, (3) cranial nerves, and (4) all remaining structures. To make the explicit segmentation easier a hierarchy of the sub-volumes was used. Accordingly, sub-volume 3 (nerves) had the highest priority, followed by sub-volume 1 (CSF including all vascular structures) and sub-volume 2 (brainstem). In this way, the previously determined segmentation results could be used as a boundary for the next step. It should be noted that only a coarse segmentation of the CSF space including the vessels and the nerve space is required, since the details are subsequently obtained with implicit segmentation.

For the 3D-visualization of the neurovascular structures, implicit segmentation in the context of direct volume rendering was used to assign individual look-up tables for color and opacity values in all four sub-volumes. Analogous to anatomical atlases, the color red was used for vessels and the color yellow for nerves. The brainstem was assigned a light grey color. Remaining structures (sub-volume 4), which do not provide relevant information for NVC syndromes, were completely transparent.

The 3D-visualizations were displayed in the used software and results were exported as image files. Results can be observed in any image management and editing program and the image files can also be transferred to the PACS.

A radiologist (1 year of experience) under the guidance of the leading software developer performed the image processing and an attending neurosurgeon (25 years of experience) rated the results.

### Image Fusion

To improve the representation of the vessels in the 3D-visualization in particular, the CISS data were registered with TOF data in a further step. Starting from corresponding point landmarks identified in both datasets, an initial rigid transformation was obtained, which provided a coarse alignment. Thereafter, the precise registration was obtained with an automatic voxel-based approach based on mutual information [22], which uses graphics hardware to accelerate the huge amount of trilinear interpolation. In order to ensure good registration, a resolution pyramid was used during the alignment procedure. In the end, the TOF data were reformatted by transformation into and interpolation within the coordinate system of the CISS data so that the arrangement, number and size of voxels in TOF and CISS data became identical. The resulting direct correspondence of pairs of voxels formed the basis for the subsequent fusion of TOF and CISS data.

**Fig. 1:**
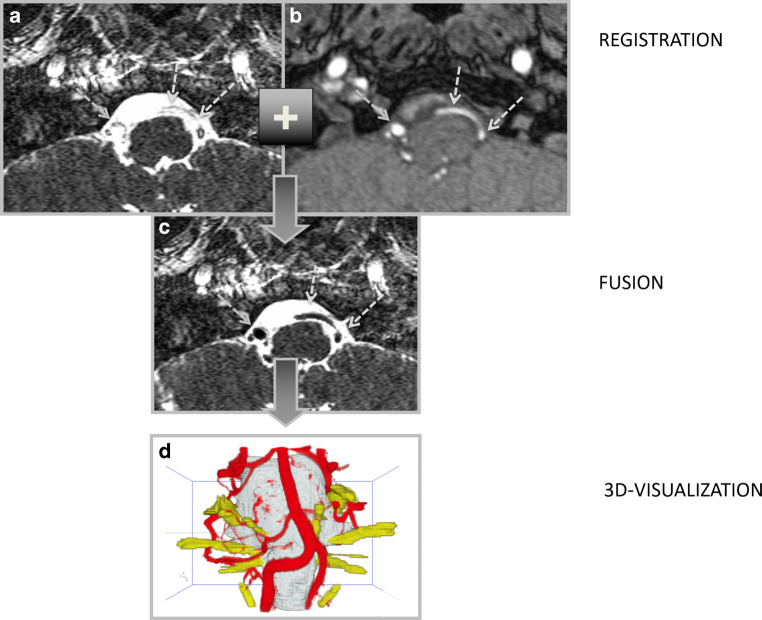
Main steps of imaging and image processing. The high resolution **a** CISS and **b** TOF data with isotropic voxel size (0.4 mm) were segmented, registered and **c** fused so that the signal information of vessels in the TOF data were transferred into the CISS data (see *arrows*). In this way, it was possible to eliminate inheriting image artifacts such as weak depiction of the vertebral artery and the PICA in the CISS data and to obtain optimized 3D-visualizations (**d**). *CISS* constructive interference in steady state, *TOF* Time-of-Flight, *PICA* posterior inferior cerebellar artery

For image fusion, voxels of hyperintense vessels in the reformatted TOF volume were segmented (extracted from the data) with volume growing. The values of these voxels were inverted and transferred to the corresponding voxel in the CISS data. As a result, vascular information of both data was combined and a new dataset with fused vascular information from TOF and CISS data was generated (3D-vis-fused) (see Fig. 1).

### Artifacts

Flow artifacts: flow-related signal variability is common in vessels with a wide lumen. In general, moving spins do not contribute to the signal formation of a given voxel at MRI, the resultant absence of the signal is known as flow void [19]. Artifacts by pulsation: artifacts by pulsation in MRI are motion artifacts caused by CSF pulsation and blood flow of the vessels. In the region of interest, the posterior fossa, mainly the basilar artery transmits the pulse wave to the CSF, which appear as artifacts. Using 3D-visualization, pulsation artifacts can be seen as a foggy cloud. Near-brainstem vessels: signal intensity values of vessels and brainstem in the original CISS data have similar values. If the course of the vessel is on the surface of the brainstem, labeling of the two structures in the step of segmentation is often difficult and may lead to incorrect labeling. This results in a decrease of image quality of the vessels in the 3D-visualizations.

### Statistical Analysis

**Fig. 2:**
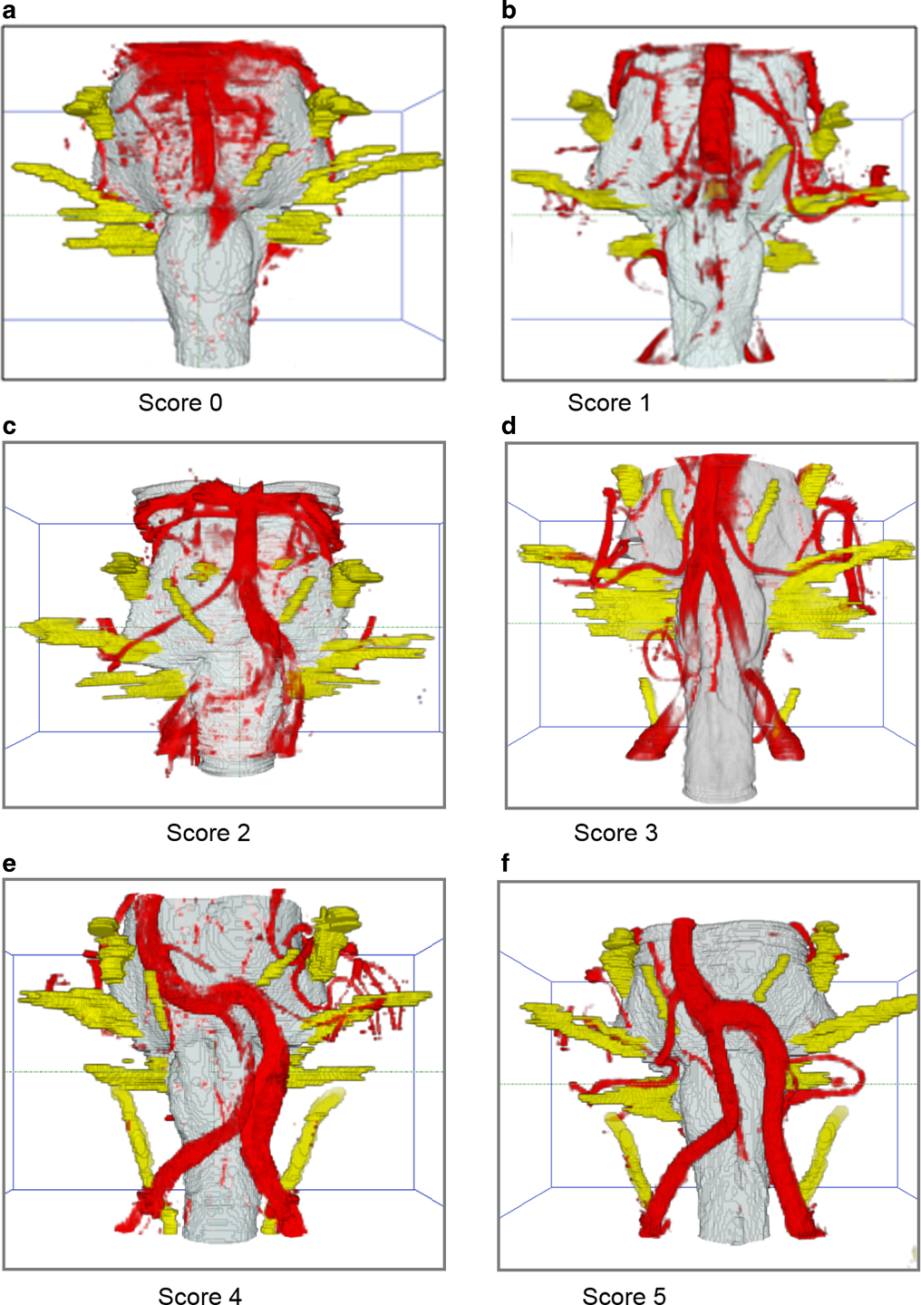
Applied ranking score on 3D-visualizations with examples. **a** Score 0: the vertebral artery and its branches are not present, the tip of basilar artery with the superior cerebellar artery are completely covered by CSF pulsation artifacts. **b** Score 1: the vertebral artery is only schematically present. **c** Score 2: the vertebral arteries are recognizable, but some parts are missing. **d** Score 3: the proximal parts of the vessels are visible. **e** Score 4: important parts of the vertebral artery are present and allow reconstructing the course of the vessel. **f** Score 5: the complete vessel is visible with the branches. *CSF* cerebrospinal fluid

A semiquantitative ranking score was used (0 insufficient quality–5 excellent quality) (details: Table 1, Fig. 2) for evaluation of the quality of imaging of the vessels in both 3D-visualizations [23]. Results of vessel image quality are expressed as means, whereas the appearance of artifacts was given as a number of affected patients. Normal distribution of data was assessed by the Levene’s test. Characteristics of 3D-visualizations before and after fusion were compared with a t-test and a χ^2^-test. Level of significance was set at a *p*-value of less than 0.05 for all tests. All calculations were performed by the software SPSS (version: Statistics 24, IBM, Armonk, NY, USA).

**Table 1:** Ranking score for the evaluation of the vessels [23]

Score	Quality
0	Missing representation of the vessel
1	Vessel is poorly or only schematically visualized
2	Vessel is recognizable but important parts are missing
3	Proximal parts of the vessel are shown
4	Important parts of the vessel are visualized
5	Complete representation of the vessel

## Results

The overall time for generation of 3D-visualization including segmentation, registration and visualization, was about 1.5 h per case. The dominating part was the semi-automatic segmentation and manual labeling (45 min). All obtained MRI data were successfully transformed through image processing into 3D-visualizations.

**Fig. 3:**
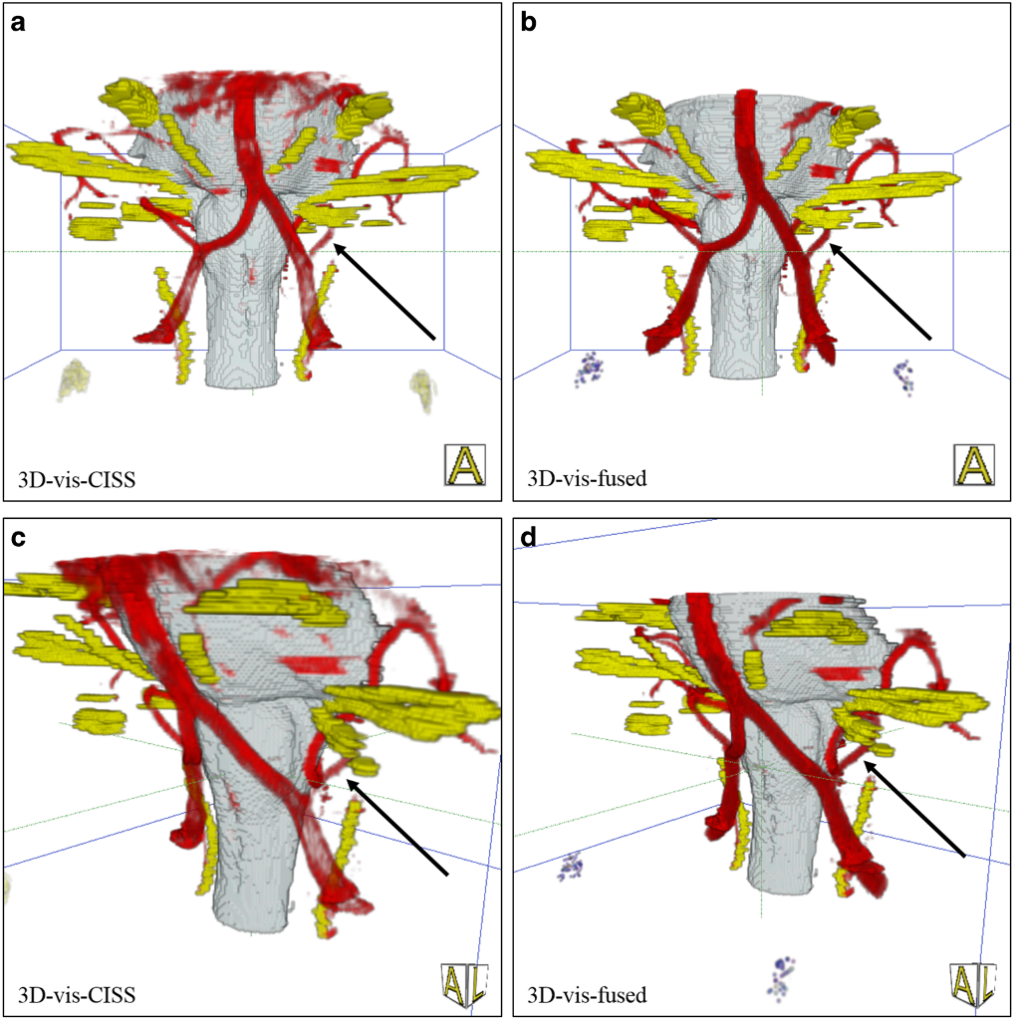
Patient with HTN and a loop of the PICA at the VLM presenting an NVC. An NVC is defined with a close relation of the vessel loop to the REZ of CN IX and X at the VLM [2]. *Arrows* indicate the NVC: in (**a**, **c**), the vessels are incompletely visualized. In the rostral part pulsation, artifacts of the CSF cover the area around the basilar artery like a fog and at the lower part around the ventrolateral medulla due to flow related artifacts parts of the left vertebral artery and the PICA are not precise. In (**b**, **d**), there is a complete representation of the NVC. *NVC* neurovascular compression, *VLM* ventrolateral medulla, *CSF* cerebrospinal fluid, *REZ* root entry zone, *CN* cranial nerve, *PICA* posterior inferior cerebellar artery

An NVC was detected on the left side in 16 patients (36%), on the right side in 7 patients (16%) and bilateral in 6 (14%) patients (example of a NVC, see Fig. 3). No NVC was seen in 15 (34%) patients.

### Evaluation of the Vessel Structures (see Table 2)

**Table 2:** Paired samples T test of image quality before and after image fusion

Vessel	Mean	Std. deviation	Std. error mean	95% confidence interval of the difference		T	Df	Sig (2-tailed)
				Lower	Upper			
B.A. CISS B.A. FUS	−0.386	1.573	0.237	−0.865	0.092	−1.629	43	0.111
L.V.A. CISS L.V.A. FUS	−1.955	1.916	0.289	−2.537	−1.372	−6.766	43	<0.001
R.V.A. CISS R.V.A. FUS	−2.227	1.987	0.3	−2.831	−1.623	−7.436	43	<0.001
L. PICA CISS L. PICA FUS	−1.432	2.005	0.302	−2.041	−0.822	−4.738	43	<0.001
R. PICA CISS R. PICA FUS	−1.227	1.903	0.287	−1.806	−0.649	−4.278	43	<0.001
L. AICA CISS L. AICA FUS	−0.341	1.38	0.208	−0.76	0.079	−1.639	43	0.109
R. AICA CISS R. AICA FUS	−0.068	0.661	0.1	−0.269	0.133	−0.684	43	0.498
Σ CISS Σ FUS	−7.705	7.07	1.066	−9.854	−5.555	−7.229	43	<0.001

*Std. deviation* Standard deviation = standard deviation of the variable, *Std. Error Mean* standard error = Standard error of sample mean, *T *t‑Statistic, *Df* degrees of freedom: *n* −1, *Sig (2-tailed)* significance = two tailed *p*-value computed using the t distribution, *B.A. CISS* basil artery constructive interference in steady state = 3D-visualization of basil artery using only CISS Data, *B.A. FUS* Basil artery fused = 3D-visualization of basil artery after image fusion, *L.V.A. CISS* left vertebral artery CISS = 3D-visualization of left vertebral artery using only CISS Data, *L.V.A. FUS* left vertebral artery fused = 3D-visualization of left vertebral artery after image fusion, *R.V.A. CISS* right vertebral artery CISS = 3D-visualization of right vertebral artery using only CISS Data, *R.V.A.* right vertebral artery fused = 3D-visualization of right vertebral artery after image fusion, *L. PICA CISS* left posterior inferior cerebellar artery CISS = 3D-visualization of left PICA using only CISS Data, *L. PICA FUS* left posterior inferior cerebellar artery fused = 3D-visualization of left PICA after image fusion, *R.PICA CISS* right posterior inferior cerebellar artery CISS = 3D-visualization of right PICA using only CISS Data, *R. PICA FUS* right posterior inferior cerebellar artery fused = 3D-visualization of right PICA after image fusion, *L. AICA CISS* left anterior inferior cerebellar artery CISS = 3D-visualization of left AICA using only CISS Data, *L. AICA FUS* left anterior inferior cerebellar artery fused = 3D-visualization of left AICA after image fusion, *R. AICA CISS* right anterior inferior cerebellar artery CISS = 3D-visualization of right AICA using only CISS Data, *R. AICA FUS* right anterior inferior cerebellar artery fused = 3D-visualization of right AICA after image fusion, *Σ CISS* Sum of CISS = D-visualization of all vessels using only CISS Data, *Σ FUS* Sum of fused = 3D-visualization of all vessels after image fusion

Comparing 3D-visualizations of CISS data (3D-vis-CISS) and 3D-visualization after registering and fusing CISS and TOF data (3D-vis-fused), we obtained a visual difference in the representation of the basilar artery.

When evaluating the representations of the vascular structures separately with respect to the left and right vertebral artery as well as the PICA, we obtained significantly higher mean values in 3D-vis-fused compared to 3D-vis-CISS (*p* < 0.001). The most frequent vessel at the VLM for NVC in patients with HTN is the PICA (see Fig. 3). Comparing the image quality of the right and left AICA in the two 3D-visualizations, we could not find any statistical significance.

However, we found a statistically significant improvement of the quality of the reproduction in the sum of all vessels (left and right vertebral artery and basilar artery, PICA and AICA on the left and right side) comparing 3D-vis-CISS and 3D-vis-fused (*p* < 0.001).

### Flow Artifacts

**Fig. 4:**
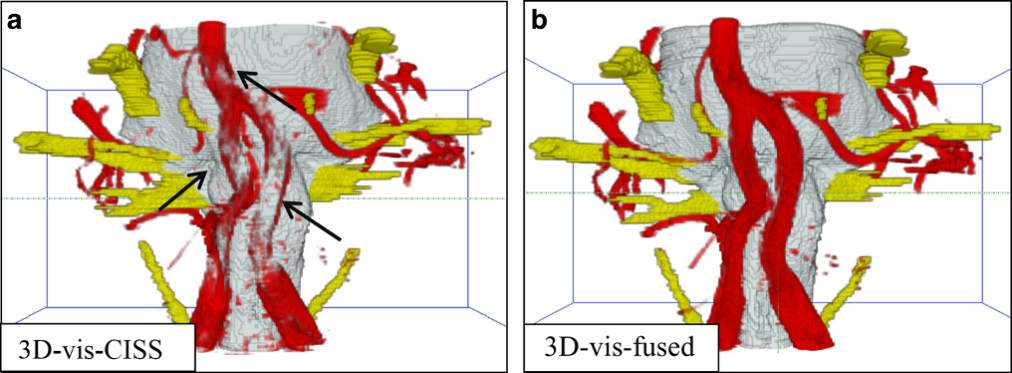
Example of flow-related signal variability in the vertebral arteries. *Arrows* point at the flow-related signal variability in the regions of the vessels

Regarding flow-related signal variability, the 3D-vis-CISS and 3D-vis-fused were compared. For the left vertebral artery in 3D-vis-CISS, 32 out of 44 patients showed vessel artifacts. In 3D-vis-fused, we found vessel artifacts on the left vertebral artery only in 2 patients. Similar results were found on the right vertebral artery, where flow artifacts could be seen in 32 patients in 3D-vis-CISS and in 4 patients in 3D-vis-fused. The basilar artery, in 3D-vis-CISS, showed flow artifacts in 10 patients and in 3D-vis-fused in 1 patient (see Fig. 4).

### Artifacts by Pulsation

**Fig. 5:**
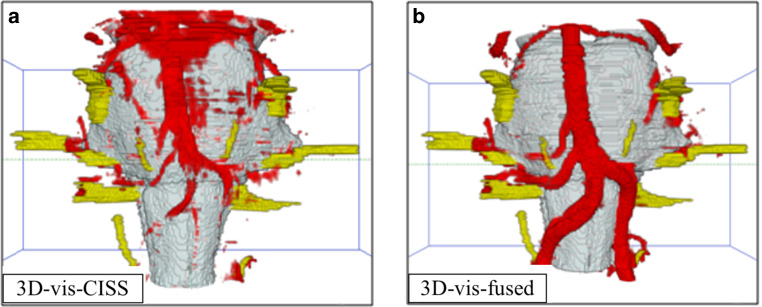
Significant improvement of 3D-visualization: **a** In 3D-vis-CISS, both vertebral arteries are incompletely visualized due to flow related artifacts. Pulsation artifacts around the basilar artery cover the upper brainstem. The left PICA is only partially represented due to contour fusion of neighboring structures. **b** In contrast, we can see a complete representation of the vessels without any pulsation artifacts in 3D-vis-fused. *CISS* constructive interference in steady state, *PICA* posterior inferior cerebellar artery

In 3D-vis-CISS, 7 patients showed pulsation artifacts of the basilar artery and 2 in the 3D-vis-fused (see Fig. 5).

### Near-Brainstem Vessels

**Fig. 6:**
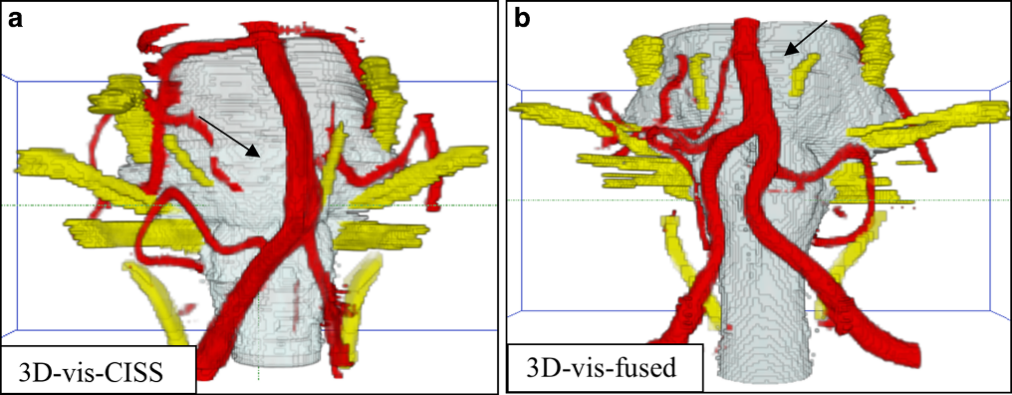
Example of a missing AICA: **a** The AICA is partially presented, and the proximal segment is not depicted (*arrow*) due to the phenomenon of contour fusion. **b** The left AICA is completely missing, probably due to a vascular anomaly in this case. *CISS* constructive interference in steady state, *AICA* anterior inferior cerebellar artery

In 3D-vis-CISS, the left and right PICA was completely reproduced in 17 patients and in 3D-vis-fused in 41 patients. Incomplete presentation of left and right PICA was seen in 3D-vis-CISS in 36 patients and in 3D-vis-fused in 29 patients. Left and right AICA were completely reproduced in 3D-vis-CISS in 32 patients and in 3D-vis-fused in 42 patients. Incomplete presentation of both AICA was seen in 3D-vis-CISS in 43 patients and in 3D-vis-fused in 35 patients (see Fig. 6). Overall, we found a statistically significant improvement of the representation of the PICA (c^2^ (1, *N* = 41) = 8.498, *p* = 0.004).

## Discussion

This study presents for the first time a highly precise 3D-visualization of the VLM in patients with essential HTN and with a special emphasis on depiction of the REZ of CN IX and X together with the surrounding vascular relationships. It describes 3D-visualization of segmented, registered and fused MR image data of the brainstem and adjacent neurovascular structures in the context of arterial HTN. The presented work quantitatively analyzed data obtained with MR imaging and image processing by comparing 3D-visualization of CISS data (3D-vis-CISS) and 3D-visualization of fused CISS and TOF data (3D-vis-fused). There is solid anatomical and physiological evidence in combination with postoperative results that treatment of essential HTN by MVD can lower blood pressure [1, 24, 25]. Until now, this treatment is not part of the clinical routine. Due to missing criteria, selection of patients is a major problem. This standardized image processing can be used for further clinical research at patients with treatment resistant HTN.

The predictive validity of MRI to identify NVC [26] that might be associated with hypertension has started a few years after the introduction of the hypothesis of neurogenic hypertension by Jannetta et al. in 1985 [1]. Several strategies have been proposed for an effective analysis of brainstem morphology in MRI [3, 7, 8, 27]. High-resolution CISS and TOF sequences are necessary tools for the assessment of neurovascular relationships in NVC. There is further consensus that patients with NVC may benefit from higher resolution and greater sensitivity of 3.0 T MRI [16]. The quality of the source images is of utmost importance [28].

In previous studies, results of the applied imaging and image processing strategies have been correlated with surgical findings in order to analyze and detect NVC in trigeminal neuralgia, hemifacial spasm and glossopharyngeal neuralgia [20, 21, 29]. We also used these strategies in patients with HTN to detect NVC at the root entry zone of CN IX and CN X [30]. It turned out that 2D-representations do not give sufficient overview of the spatial relationships of nerves and vessels [7]. According to the literature, the presence of NVC is considered in image analysis if a vessel is touching a nerve without visible layer of CSF inbetween [13].

The quality of the generated 3D-visualizations was objectively determined by assessing artifacts of imaging and evaluating the vascular reproductions. The data show that the fusion of the two MR data CISS and TOF leads to a reliable 3D-visualization of the anatomical course of CN IX and CN X and at the same time to a significantly increased quality of the vessel presentation. Thereby, the precise identification of the nerve-vessel relationships is available (see Fig. 3).

We were able to assess a 39.66% increase in the quality of the vessel presentation for 3D-vis-fused compared to 3D-vis-CISS, see Table 2. A detailed analysis of the vessels in the prepontine cistern revealed that the mean vessel quality was statistically higher in 3D-vis-fused than in 3D-vis-CISS, with exception of the AICA and basilar artery. In the case of an anatomical variant of a missing AICA, we evaluated this variability with the score 0 (see Fig. 6). This might explain the small difference in the two 3D-visualizations regarding the quality of vessel reproduction of the AICA. In addition, a reduction of measurable pulsation and flow artifacts was achieved with 3D-vis-fused. Flow-related artifacts occur mainly due to the hyperintense signal within vessels with large lumen in CISS data. This physical attribute leads to a reduced image quality of the vessels in 3D-vis-CISS. In the affected vessels, we observed a reduction of flow artifacts in 30 patients for the left vertebral artery, in 28 patients for the right vertebral artery and in 9 patients on the basilar artery in 3D-vis-fused as compared to 3D-vis-CISS. Adequate reproduction of vessels in 3D-visualizations has been shown to be crucial for the detection of NVC [7]. Artifacts from CSF pulsation overlay the vessels in 3D-visualizations as they appeared like a cloud of dots around the vessels in the CSF area. This affects mainly the basilar artery due to the strong pulse wave transmitted to the CSF in the prepontine cistern. In 3D-vis-CISS, 7 patients showed CSF pulsation artifacts around the basilar artery. After image fusion, pulsation artifacts were eliminated in 5 cases and remained much weaker in 2 cases in 3D-vis-fused. Combination of poor vessel reproduction due to flow artifacts and pulse wave artifacts are the major causes for indeterminate 3D-visualizations thus obscuring the presence of NVC (see Fig. 5). Vessels that closely followed the surface of the brainstem could not always be delineated due to contour fusion [20]. This physical aspect is particularly evident in the course of the PICA and AICA as they often run along the surface of the brainstem. A detailed analysis of these vessels showed a reproduction increase of 144% for PICA and 31% for AICA in 3D-vis-fused compared to 3D-vis-CISS (see Figs. 3 and 6).

There were limitations to our study. The applied image processing strategy was used on a small number of patients. Only 44 patients with refractory HTN entered the study; however, we are able to present an approach for the detection of NVC of CN IX and CN X for further clinical examinations and investigations. Although the evaluation of the 3D-representations is easy and comprehensible, expert knowledge about the complex anatomy of neurovascular relationship is still needed in the currently semiautomatic image processing. In future further automation of image processing is necessary. Since there are no established criteria to indicate surgical exploration of the posterior fossa and microvascular decompression in patients with essential HTN, 3D-visualization of this region based on image data may help to find candidates who might benefit from decompression.

Sindou et al. recently applied an MRI protocol to detect NVC at CN VII and CN X−IX in a prospective study of 48 patients with hemifacial spasm associated with essential hypertension. They used high-resolution 3D T2 weighted, 3D gadolinium-enhanced T1 weighted and 3D TOF imaging [25]. This protocol was suggested by Leal et al. and compared to surgical findings in patients with trigeminal neuralgia who underwent MVD [13]. Both studies were based on 2D-visualizations with slice images. To evaluate the hypothesis of an association between NVC and HTN, we believe our proposed 3D-visualization strategy presents a new standard for research. Akimoto et al. made a first approach of 3D-visualization in patients with trigeminal neuralgia suggesting that the 3D-presentations are very useful for preoperative simulations and the decision whether to perform surgery [31]. Haller et al. presented a study of imaging of NVC syndromes, where CISS and TOF data were fused; however, they used only 2D-visualizations using slice images [32]. In general, we can see that NVC is common in asymptomatic patients undergoing MRI. Several factors must be considered to regard a neurovascular contact as symptomatic. The exact anatomic knowledge of the position and morphology of the REZ and the irritating vessel is of fundamental importance for interpretation of neurovascular findings in these cases. With the method of image processing introduced by our research group [15], whose 3D-representations have been correlated with surgical findings in several studies on neurovascular compression syndromes [7, 20, 21], we present a robust and fast approach to generate 3D-visualizations in the context of HTN. With our method, it is possible to verify in vivo the three distinct types of NVC at the VLM [2].

## Conclusion

This study focused on the development, analysis and introduction of the technique for 3D-visualization of neurovascular compression (NVC) at the ventrolateral medulla (VLM) in hypertension (HTN).

The presented method of imaging and image processing delineates the anatomical course of vessels and nerves in the posterior fossa in an accurate and clear way. 3D-visualization can detect and analyze NVC at the VLM in patients with HTN. It allows noninvasive examinations.

In summary, we present a standardized, fast and robust strategy to obtain a detailed anatomical view on the neurovascular relationships at the VLM. This standardized tool provides validated non-invasive 3D-representations of the VLM for further clinical research in treatment resistant HTN.

## References

[1] Jannetta PJ, Segal R, Wolfson SKJ (1985). Neurogenic hypertension: etiology and surgical treatment. I. Observations in 53 patients. Ann Surg.

[2] Naraghi R, Gaab MR, Walter GF, Kleineberg B (1992). Arterial hypertension and neurovascular compression at the ventrolateral medulla. A comparative microanatomical and patholgical study. J Neurosurg.

[3] Hohenbleicher H, Schmitz SA, Koennecke HC, Offermann R, Offermann J, Zeytountchian H (2001). Neurovascular contact of cranial nerve IX and X root-entry zone in hypertensive patients. Hypertension.

[4] Johnson DR, Coley SC, Brown J, Moseley IE (2000). The role of MRI in screening for neurogenic hypertension. Neuroradiology.

[5] Legrady PVE, Bajcsi D, Sonkodi S, Barzo P, Abraham G (2008). Neurovascular pulsatile compression and neurosurgical decompression of the rostral ventrolateral medulla in medically resistant hypertensive patients. Kidney Blood Press Res.

[6] Naraghi R, Geiger H, Crnac J, Huk W, Fahlbusch R, Engels G, Luft FC (1994). Posterior fossa neurovascular anomalies in essential hypertension. Lancet.

[7] Naraghi R, Hastreiter P, Tomandl BF, Bonk A, Huk W, Fahlbusch R (2004). Three dimensional visualization of neurovascular relationship in the posterior fossa—technique and clinical application. J Neurosurg.

[8] Sagelitz SA, Gaab MR (2002). Investigations using magnetic resonance imaging: is neurovascular compression present in patients with essential hypertension?. J Neurosurg.

[9] Thuerl C, Rump LC, Otto M, Winterer JT, Schneider B, Funk L, Laubenberger J (2001). Neurovascular contact of the brain stem in hypertensive and normotensive subjects: MR findings and clinical significance. AJNR Am J Neuroradiol.

[10] Zizka J, Ceral J, Elias P, Tintera J, Klzo L, Solar M, Straka L (2004). Vascular compression of rostral medulla oblongata: prospective MR imaging study in hypertensive and normotensive subjects. Radiology.

[11] Stobo DB, Lindsay RS, Connell JM, Dunn L, Forbes KP (2011). Initial experience of 3 Tesla versus conventional field strength magnetic resonance imaging of small functioning pituitary tumours. Clin Endocrinol (Oxf).

[12] Ross JS (2004). The high-field-strength curmudgeon. AJNR Am J Neuroradiol.

[13] Leal PR, Hermier M, Froment JC, Souza MA, Cristino-Filho G, Sindou M (2010). Preoperative demonstration of the neurovascular compression characteristics with special emphasis on the degree of compression, using high-resolution magnetic resonance imaging: a prospective study, with comparison to surgical findings, in 100 consecutive patients who underwent microvascular decompression for trigeminal neuralgia. Acta Neurochir (Wien).

[14] Tanrikulu L, Hastreiter P, Richer G (2008). Virtual neuroendoscopy: MRI-based three-dimensional visualization of the cranial nerves in the posterior cranial fossa. Br J Neurosurg.

[15] Hastreiter P, Naraghi R, Tomandl BF, Bauer M, Fahlbusch R (2002). 3D-visualization and registration for neurovascular compression syndrome analysis.

[16] Garcia M, Naraghi R, Zumbrunn T, Rösch J, Hastreiter P, Dörfler A (2012). High-resolution 3D-constructive interference in steady-state MR imaging and 3D time-of-flight MR angiography in neurovascular compression: a comparison between 3T and 1.5T. AJNR Am J Neuroradiol.

[17] Hastreiter P (1999). Registrierung und Visualisierung medizinischer Bilddaten unterschiedlicher Modalitäten.

[18] Mancia G, Fagard R, Narkiewicz K (2013). 2013 ESH/ESC Guidelines for the management of arterial hypertension: the Task Force for the management of arterial hypertension of the European Society of Hypertension (ESH) and of the European Society of Cardiology (ESC). J Hypertens.

[19] Pandey S, Hakky M, Kwak E, Jara H, Geyer CA, Erbay SH (2013). Application of basic principles of physics to head and neck MR angiography: troubleshooting for artifacts. Radiographics.

[20] Naraghi R, Tanrikulu L, Troescher-Weber R, Bischoff B, Hecht M, Buchfelder M (2007). Classification of neurovascular compression in typical hemifacial spasm: three-dimensional visualization of the facial and the vestibulocochlear nerves. J Neurosurg.

[21] Tanrikulu L, Hastreiter P, Dörfler A, Buchfelder M, Naraghi R (2015). Classification of neurovascular compression in glossopharyngeal neuralgia: three-dimensional visualization of the glossopharyngeal nerve. Surg Neurol Int.

[22] Teßmann M, Eisenacher C, Enders F, Stamminger M, Hastreiter P (2008). GPU Accelerated Normalized Mutual Information and B-Spline Transformation.

[23] Hastreiter P, Dodenhoeft N, Troescher-Weber R, Hastreiter L, Buchfelder M, Naraghi R (2006). Modern strategies of image processing for neurovascular compression in arterial hypertension.

[24] Geiger H, Naraghi R, Schobel HP, Frank H, Sterzel RB, Fahlbusch R (1998). Decrease of blood pressure by ventrolateral medullary decompression in essential hypertension. Lancet.

[25] Sindou M, Mahmoudi M, Brînzeu A (2015). Hypertension of neurogenic origin: effect of microvascular decompression of the CN IX-X root entry/exit zone and ventrolateral medulla on blood pressure in a prospective series of 48 patients with hemifacial spasm associated with essential hypertension. J Neurosurg.

[26] Boogaarts HD, Menovsky T, de Vries J, Verbeek AL, Lenders JW, Grotenhuis JA (2012). Primary hypertension and neurovascular compression: a meta-analysis of magnetic resonance imaging studies. JNS.

[27] Ceral J, Zizka J, Elias P, Solar M, Klzo L, Reissigova J (2007). Neurovascular compression in essential hypertension: cause, consequence or unrelated finding?. J Hum Hypertens.

[28] Tomandl BF, Hastreiter P, Rezk-Salama C, Engel K, Ertl T, Huk WJ (2001). Local and remote visualization techniques for interactive direct volume rendering in neuroradiology. Radiographics.

[29] Tanrikulu L, Hastreiter P, Troescher-Weber R, Buchfelder M, Naraghi R (2007). Intraoperative three-dimensional visualization in microvascular decompression. J Neurosurg.

[30] Hastreiter P, Naraghi R, Tomandl BF, Bonk A, Fahlbusch R (2003). Analysis and 3-dimensional visualization of neurovascular compression syndromes. Acad Radiol.

[31] Akimoto H, Nagaoka T, Nariai T, Takada Y, Ohno K, Yoshino N (2002). Preoperative evaluation of neurovascular compression in patients with trigeminal neuralgia by use of three-dimensional reconstruction from two types of high-resolution magnetic resonance imaging. Neurosurgery.

[32] Haller S, Etienne L, Kövari E, Varoquaux AD, Urbach H, Becker M (2016). Imaging of neurovascular compression syndromes: trigeminal neuralgia, hemifacial spasm, vestibular paroxysmia, and Glossopharyngeal neuralgia. AJNR Am J Neuroradiol.

